# The Motherhood Penalty of Immigrants in France: Comparing the Motherhood Wage Penalty of Immigrants From Europe, the Maghreb, and Sub-Sahara With Native-Born French Women

**DOI:** 10.3389/fsoc.2022.748826

**Published:** 2022-03-30

**Authors:** Noa Achouche

**Affiliations:** ^1^The Gender Studies Program, Bar-Ilan University, Ramat Gan, Israel; ^2^The Institute for Immigration and Social Integration, Ruppin Academic Center, Hefer Valley, Israel

**Keywords:** motherhood penalty, immigration, ethnicity, motherhood ideology, intersectionality, self-selection, family investment hypothesis, immigration in France

## Abstract

To date, relatively few studies analyzed the motherhood penalty as experienced by immigrant women. The principal objective of this research is to establish whether the negative effect of motherhood on wages is higher for immigrants than it is for the native population; and how this effect may vary across different immigrant regions of origin. Using data from the Enquête Revenus Fiscaux et Sociaux from 2009 to 2012 (INSEE, 2009–2012)^1^, a series of linear regression models were calculated to examine whether the effect of motherhood on wages differs for immigrant women and native women; and to what extent this effect varies across different immigrant origin groups. Specifically, this study explores the effect of motherhood on immigrant labor market integration in France from three regions of origin, as compared to native French women: immigrants from sub-Saharan Africa, the Maghreb (Algeria, Tunisia, and Morocco), and from European countries (each and every region of origin is considered separately in comparison to native French women). The results revealed substantial differences in the motherhood penalty between the different regions of origin and assert the existence of an especially pronounced motherhood penalty for mothers from the Maghreb. Given the gap in the research with regards to the cost of motherhood for immigrants in the labor market of the host country, this research sheds light on specific mechanisms influencing the integration patterns of immigrant women. Moreover, by choosing France, which is one of the main immigration destinations in Europe, and a country where the motherhood penalty for the native population is almost non-existent, this study provides a new perspective on the intersection of motherhood, immigration, and region of origin in the immigrants' labor-market integration process.

## Introduction

Scholars have identified abundant evidence explaining differences in the integration of immigrants in host countries from various region of origin. Academic literature points out the difficulties, that immigrants face in finding suitable and rewarding employment in the labor market of a variety of host countries (Chiswick, 1978, 1980; Borjas, 1987, 1992, 1995; Gorodzeisky and Semyonov, 2011).

The ethnicity or region of origin of immigrants has been underscored as a one of the factors affecting their integration. As such, immigrant integration into the labor market of the host countries differs across ethnic and gender groups. More specifically, immigrants from less economically developed countries tend to face greater difficulties in finding suitable employment compared to other immigrant groups (e.g., Raijman and Semyonov, 1997; Heilbrunn et al., 2010; Pichler, 2011). Research has further shown that regardless of region of origin, immigrant women experience greater difficulties than immigrant men in the labor market of the host society (Raijman and Semyonov, 1997; Logan and Rivera Drew, 2011; Fleischmann and Höhne, 2013). One aspect that remains relatively unstudied is the specific cost of motherhood for immigrant women compared to their non-immigrant peers in the host country.

Various research suggests that the gender gap between men and childless^2^ women in the general population has shrunk with regard to employment and earnings (Budig and England, 2001; England, 2005). However, the gap between women with children and those without children seems to have remained across most Western countries (Budig and England, 2001; England, 2005; Budig et al., 2012, 2016; Halldén et al., 2016; Cukrowska-Torzewska, 2017; Cukrowska-Torzewska and Lovasz, 2020). This gap is known in the literature as the “motherhood penalty.” The motherhood penalty and the integration patterns of immigrants have been the subject of numerous studies taken separately, but much less attention has been focused on considering the relationship between them. Consequently, it has not been established whether the negative effect of motherhood on wages would be higher among immigrants than among the native population and whether this effect differ by region of origin^3^.

In order to fill this gap in the literature, this study aims to answer the following questions: (1) (a) Does a woman's immigration status and motherhood affect her income, and if so, to what extent? (b) Does the effect of motherhood on income differ for immigrant women compared to native women? (c) Does the effect of motherhood on income differ across origin groups of immigrants^4^?

The case of economic integration of immigrant women coming from three groups of origin is examined: immigrants from sub-Saharan Africa (Senegal, Mali, Cote d'Ivoire, Cameroon); immigrants from the Maghreb (Algeria, Tunisia, and Morocco); and immigrants from several European countries. Each of the three regions of origin will be considered separately in relation to native-born French women^5^.

France has a long history of immigration and a considerable immigrant population (12%: OECD/European Union, 2018). The composition of the immigrant population into relatively large groups of immigrants facilitates the identification of specific immigration patterns and cultural differences among these groups. Finally, studies on the motherhood penalty in France have found it to be nearly non-existent there in terms of wages (Budig et al., 2012, 2016), with virtually no penalty for mothers with one or two children—the penalty begins to be manifested only after the third child. The lack of a motherhood penalty in France has led to a paucity of research on the subject exclusively within the French context; the identification of such an effect among France's immigrant population, and especially within specific groups, may lead to a renewed interest encouraging further research on the topic.

The literature on gender has often argued that the intersectional identities of some groups of women generates additional disadvantages in the labor market beyond those incurred by the identity of being a woman alone (Browne and Misra, 2003; Glauber, 2008; Mandel, 2012). In this study, I argue that understanding the significance of the intersection between gender, ethnicity, and motherhood for different groups of women is crucial in order to understand the perpetuation of gender inequality. Moreover, earlier lines of research in France focused largely on the experiences of native women, and subsequently lacked an intersectional approach. Thus, in expecting a divergent effect of motherhood among minorities, this study proposes that the intersectional identities of immigrant mothers can have important outcomes for their success in the labor market.

## Chapter 1: Theoretical Background

### Gender and Immigration

According to the “classic assimilation model,” immigrants can face disadvantages in the first years after immigration due for the most part to limited familiarity with the host country's language, lack of educational and cultural resources, limited social networks and restricted access to information. Due to these limitations, upon arrival in the host society immigrants often accept lower paying jobs that do not match their skills and human capital attributes. Earnings of immigrants are therefore lower than among their native-born counterparts. Nevertheless, with the passage of time, the economic disadvantages of immigrants tend to decline and their advantages even surpass the native-born population in some cases (Chiswick, 1980).

Early studies on the assimilation trajectory of immigrant women argued that in the first years of immigration, women tend to have high levels of employment that decrease with time in the host country (Chiswick, 1980; Long, 1980). Women who immigrate with a partner or a family are assumed to be “tied movers.” The term “tied migration” refers to migration resulting from a decision taken by one member of a couple or family to immigrate along with their partner in order to maximize family earning potential, irrespective of individual earning potential (Mincer and Polachek, 1978). Since women are assumed to immigrate due to family imperatives rather than independent economic aspirations, immigrant women work in the host country only to support their partner's career. However, once their partner reaches an improved financial status in the host country, women tend to decrease their economic participation. Therefore, the economic integration course of immigrant women may be less linear compared to that of immigrant men (Mincer and Polachek, 1978; Chiswick, 1980; Long, 1980; Baker and Benjamin, 1997).

Whereas, the incentive of childless women to immigrate is likely to stem from a motivation to better their own individual economic prospects, women with children, by contrast, are more likely to be tied movers. However, since family responsibilities are traditionally held by women, the mere existence of children within a family is the primary family responsibility for both native and immigrant women. Immigrant women often leave behind their network of family and friends that usually aids in the support of family responsibilities. In addition, immigrant women often lack an established professional network. Thus, immigrant women with children may suffer disproportionately lower labor market prospects compared to childless immigrant women and native mothers^6^. Consequently, immigrant women with family responsibilities are likely to face greater hardship in finding suitable employment, resulting in reduced interaction with the local population and therefore fewer opportunities to learn the local culture and language. The integration process will be slower, and employment prospects will thus be reduced (Boyd, 1984; Raijman and Semyonov, 1997; Hondagneu-Sotelo, 2003; Pessar, 2003).

### Differences Between Natives and Immigrants: The Motherhood Penalty

Although the economic gap between men and women has decreased in the past three decades, the gap between women with children and those without children has remains a feature in most Western countries (Budig and England, 2001; Avellar and Smock, 2003; Gough and Noonan, 2013; Levanon and Grusky, 2016). The cost of motherhood encompasses multiple elements—not only is there discrimination against mothers by employers (Benard et al., 2007), but career interruption, too, results in the loss of experience and employment tenure (Budig and England, 2001; England et al., 2016; Killewald and García-Manglano, 2016) and fewer opportunities to accumulate human capital (Miller, 2011; Kahn et al., 2014; Williams and Dempsey, 2014; Gough, 2017). Some scholars also argue that motherhood may also lead to lower job performance, resulting from the double shift that these women are burdened with, women consequently choosing mother-friendly segregated jobs, trading wages and occupational prestige for more compatible arrangements (Becker, 1991; Budig and England, 2001).

The disadvantages faced by mothers in terms of income differ considerably by country, class, marital status, ethnicity, country of origin, race, and labor market context (Raijman and Semyonov, 1997; Browne and Misra, 2003; Glauber, 2008; Mandel, 2010; England et al., 2016). The wage penalty for mothers ranges from 0 to 1% in Israel and Belgium (Davies and Pierre, 2005; Budig et al., 2012) to as much as 18% per child in the Netherlands or West Germany (Budig et al., 2012, 2016) even after controlling for background variables and selection.

Although all mothers—immigrant and native alike—may suffer from the negative effect of motherhood on wages, immigrant mothers are likely to suffer further costs due to their immigration status. First, immigrants may have difficulties in the first years after immigration due to limited knowledge of the language in the host country and its local culture and norms. Moreover, immigration to a new country typically involves interruption of prior work in the country of origin, which, taken together with the immigration itself incurs loss of established personal or professional connections. These women will need to build an entirely new professional network in the host country, an endeavor for which the presence of children constitutes an added disadvantage to both immigration and motherhood independently.

The combination of these obstacles will negatively affect their professional trajectory in comparison with that of native mothers; when intersected with the general motherhood penalty, it may create an additional disproportionate cost compared to the one paid by immigrant women with no children and native mothers^7^.

At this point, we can formulate the following hypotheses on the relationship between immigrant status, motherhood, and economic integration into the host country's labor market:

*Hypothesis 1*—*refers to the labor market outcomes of immigrant mothers in the host country's labor market, compared to immigrant women without children:*
  (H1a) Immigrant mothers will have lower wages, compared to immigrant women without children.
*Hypothesis 2*—*refers to the labor market outcomes of the immigrant mothers in the host country's labor market, compared to native mothers:*
  (H2a) Immigrant mothers will have lower wages, compared to native mothers.
  (H2b) The negative effect of motherhood on wages of immigrant women will be higher than such an effect on wages of native women.

### Differences in Country of Origin in Selection of Immigrant Women

The literature indicates that immigration costs can vary between different geo-cultural immigrant groups. The cost of immigration fluctuates in relation to the immigrant's country of origin and ethnic background, for both men and women (Boyd, 1984; Blau et al., 2011; Khoudja and Fleischmann, 2015; Ala-Mantila and Fleischmann, 2018). When immigrating to a developed country, immigrants from poor, less developed countries face more hurdles than immigrants from developed countries, resulting in a higher loss of actualization of their human capital (Friedberg, 2000; Reitz, 2001; Rumbaut and Portes, 2001).

Immigration may offer better chances for some women than those available in their country of origin; when emigrating, however, women from less developed countries of origin may face more disadvantages than women emigrating from more developed ones (Boyd, 1984; Raijman and Semyonov, 1997; Antecol, 2000; Blau et al., 2011; Gorodzeisky and Semyonov, 2011; Ala-Mantila and Fleischmann, 2018). However, women from different regions of origin may be positively or negatively selected according to their motivation for immigration.

As such, according to the family investment model (Baker and Benjamin, 1997), women who are tied movers from more economically developed countries will be more likely to have partners who are positively selected in the host country's labor market^8^. It is thus less probable that these women will be in a position in which their economic input is vital for the family's survival, and therefore will be employed at a lower rate. However, when employed, it may be by choice and thus more likely to be at higher wages and occupational status. On the other hand, women with children from poor, less developed countries may have a high rate of employment. However, since they are likely to be working in order to compensate for their partner's insufficient earnings and to ensure family survival, they may be obliged to accept whatever job is available to them, possibly at lower wages and occupational status.

Cultural differences, gender norms and motherhood ideology can vary across countries of origin and geo-cultural backgrounds (Inglehart and Norris, 2003; Idema and Phalet, 2007; Diehl et al., 2009). Studies in the United States, for example, have shown that the motherhood penalty was lower for Afro-American women (Gough and Noonan, 2013). Afro-American women may be in extreme need of employment due to their precarious economic conditions and their relatively low marriage rates, but also because of low earnings and work instability of racial minority men (Glauber, 2008, 2018; Gough and Noonan, 2013). Moreover, African culture has always encouraged mothers to be responsible for the economic wellbeing of their children and family. These work and motherhood practices are grounded in the historical, economic, and cultural traditions of African women (Branch, 2011; Dow, 2016).

In contrast, strong religious commitment, especially Islam, has been presented as one of the main factors contributing to the cultural preservation of traditional gender-role behaviors, and individuals with strong religious commitment levels are less likely than their secular counterparts to hold egalitarian gender-role attitudes (Idema and Phalet, 2007; Diehl et al., 2009). A large number of studies have found that Muslim immigrants face the largest disadvantages, even when compared to other migrants or immigrants from the same countries of origin that are not Muslim (Silberman et al., 2007; Khattab and Modood, 2015). In addition, Muslim women (immigrant or native) have lower levels of employment than women from other religious groups, even after controlling for human capital and family status characteristics. This is true in the United Kingdom (Connor and Koenig, 2015; Abdelhadi and England, 2019), Germany (Diehl et al., 2009), the Netherlands (Khoudja and Fleischmann, 2015), Canada (Reitz et al., 2015), India (Klasen and Pieters, 2015), and Malaysia (Amin and Alam, 2008).

Due to low labor force participation rates of women and traditional gender-role attitudes in traditional countries of origin, women from these countries may have greater difficulties entering the labor market of the host country. Yet, the barriers that they have to overcome to be active in the host country's labor market may cause them, as a result, to be a positively selected group with high human capital, with higher labor market outcomes than women who did not have to face such obstacles (Borjas, 1987, 1992; Stier and Tienda, 1992; Reitz, 2001). Thus, a positive selection of women from traditional countries of origin into the host country's labor market can occur. On the other hand, immigrant mothers belonging to groups with modest resources and with low levels of cultural and religious similarity to the host country may face a higher level of discrimination and tend to experience a greater motherhood penalty compared to mothers who immigrated from countries of origin with a high level of cultural similarity^9^.

### Host Country Characteristics—The Context of France

Research studies have repeatedly demonstrated that the motherhood penalty in France is nearly non-existent (Budig et al., 2012, 2016). The wage penalty for one or two children does not exist at all, with the penalty beginning manifestation only after the third child, depending on the estimation method (Davies and Pierre, 2005). The relatively low motherhood penalty in France is typically explained by family-friendly policies such as free daycare facilities open all day long, and accommodating for children as young as 3 months old (Cukrowska-Torzewska, 2017; Lucifora et al., 2017). Moreover, the combination of paid work and family is socially accepted in France.

France is a country with a long history of immigration (Noiriel, 1988; Algan et al., 2012; Pailhé, 2015). In the years following World War II, France adopted an organized and controlled immigration policy. This rather exclusionary immigration policy accepted immigration primarily from former French colonies, Turkey, and European countries. Immigrants, mostly men, came to France from Turkey, Spain, Portugal, Morocco, and Algeria. After 1974, the government limited immigration to family reunification alone, and to specific work permits stemming from employer requests (Perrin-Haynes, 2008). Later, the formation of the European Union (EU) in 1993, establishing a single market accompanied by the free flow of labor, and further EU expansion in 2004, led to a massive migration movement within EU borders.

The majority of immigrants from former French colonies have a good command of the French language since it was the official language for all French colonies. Despite this linguistic advantage, the majority of France's immigrant populations are manual workers who suffer from poor employment conditions (Constant and Zimmermann, 2005).

Different immigrant origin groups in France experience divergent paces of integration. Studies in France comparing Portuguese, Asian and Turkish immigrant communities with the North African immigrant communities have shown that while the first three groups are less assimilated culturally, they also experience less racism and are more dynamic economically than the North African community (Dubet, 1989; Alba and Foner, 2016). The public debate on immigration focuses almost exclusively on Islam, a religion perceived by mainstream French society as a threat to basic laic values (Silberman et al., 2007; Thomson and Crul, 2007; Foner and Alba, 2008; Koopmans, 2016). As such, religion in France is considered a factor delaying integration and the French native population has come to associate Islam with problematic integration; anti-immigration sentiments are becoming more pronounced and have taken the form of an essentialization of religion and anti-immigration sentiment (Foner and Alba, 2008; Blommaert et al., 2014).

We can assume that the magnitude of the impact of motherhood on immigrant integration in France differs by region of origin, as the regions are characterized for the most part by different levels of economic development, gender ideology, levels of traditionalism with respect to gender norms and cultural and religious similarity to the native population. Women immigrating from European countries are likely to have better labor market outcomes than immigrant from Maghreb and sub-Saharan Africa, due to a number of reasons. First, European countries share overall similar income distribution patterns with France (Frikey et al., 2004; Silberman et al., 2007; Perrin-Haynes, 2008). Immigrants from European countries are likely to share similar cultural values and religious backgrounds with French society, including beliefs regarding gender-role attitudes. As a result, they are likely to face relatively less discrimination from French employers compared to other immigrant groups. Immigrant mothers from Europe, however, may conceivably be in less immediate need of additional income when immigrating with a partner who possesses high human capital transferability. Yet due to the high selectivity of these women into the French labor market, when employed, they may have higher income compared to immigrant mothers from the Maghreb and sub-Saharan Africa.

Immigrant women from the Maghreb are likely to bear the highest penalty of all groups, regardless of motherhood status, due to high inequality in income distribution in their countries of origin and the fact that their cultural values may be very different from French natives. Gender-role norms and attitudes of women from the Maghreb are likely to be very different from those of natives, especially since they come primarily from highly traditional and religious Muslim countries (Diehl et al., 2009; Röder and Mühlau, 2014). Immigrant women from the Maghreb, therefore, can be expected to be a disadvantaged group, especially when they have children. When employed, however, it is unclear whether these women will have low income compared to immigrant mothers from sub-Saharan Africa, European countries, and native mothers, or, on the contrary, high income as a result of the possible high selection of these women into the French labor market.

Due to the low level of development in their countries of origin, immigrants from the sub-Sahara may experience the low transferability of their human capital, and thus be obliged to accept occupations that do not match their qualifications. As a result, we may observe lower income for women from sub-Saharan Africa, who may prefer to work under lower conditions that nevertheless subsidize occupational adequacy with essential income generation.

At this point, we can formulate the following hypotheses about the interaction between country of origin and the labor market outcome of mothers in France:

*Hypothesis 3*—*refers to the economic outcome of immigrant mothers by region of origin, compared to native mothers in the French labor market*.
  (H3a) The wages of an immigrant mother from Europe in the French labor market are higher than the wages of an immigrant mother from the Maghreb or from sub-Saharan Africa.

A positive selection of immigrant women from traditional and conservative regions of origin^10^ may arise in the French labor market, and thus:

(H3b) The income of an immigrant mother from the Maghreb in the French labor market are higher than the income of an immigrant mother from European countries or from sub-Saharan Africa, due to the high selectivity of these women into the French labor market.

## Chapter 2: Data Variables and Methods

### Data and Variables

The Data was taken from the French “Enquête des Revenus Fiscaux et Sociaux (ERFS),” 2009–2012 (ERFS, INSEE, 2009, 2010, 2011, 2012), the French labor force census, providing information on more than 600 variables at the individual and household level. In order to analyze a sample including a sufficient number of cases, I pooled 4 years of data (2009-2012). The pooled sample included information on 66,539 native women and 10,983 foreign-born women (first-generation immigrants) between 18 and 50 years old.

The dataset includes information on gender, age, marital status as well as cohabitation, the number of children at home under 18 years, the number of children at home under 6 years, education, occupation, employment status, income, weekly working hours, country of birth^11^ and years since immigration^12^.

#### Variables

This study focuses on income as the dependent variable. The income level is calculated as the logarithmic transformation of annual wages. Only women working more than 5 h a week and earning between €10 and €100,000 annually have been included, conditions that represent 99% of the working population. I noticed that most self-employed women do not take a salary and 80% of them reported a €0 annual income; I chose to omit them from the models.

The main independent variables relate to motherhood and immigration status^13^.

The literature suggests that the presence of young children is specifically related to the motherhood penalty since young children require more care and cannot be left alone during working hours (Anderson et al., 2003). On the other hand, the literature also indicates that the motherhood penalty has lifetime consequences on women's employment and the effect of motherhood can be identified long after children have passed the early childhood stage (Budig and England, 2001; Benard et al., 2007). Although the literature provides sufficient evidence on the effect of both the age and number of children on the motherhood penalty, the small variance in the number of children among women in France created multicollinearity, with inconsistent and insignificant results. Moreover, although France provides extended family policies such as free daycare facilities for children from a very young age, gender norms of certain immigrant groups can shape attitudes associated with childcare, thus mothers may be expected to provide primary care for their young children despite those policies. Older children may not require such an extended level of care because older children can provide for their own needs more easily and have more agency. I decided therefore to take the age of children into account instead of the number of children^14^. Nevertheless, since norms can shape gender-role attitudes associated with childcare, I expect the effect of young children to fluctuate across immigrant groups, depending on the origin group to which the women belong^15^.

The variable related to motherhood is calculated using two different sets of dummy variables including three categories and indicating whether the respondent has children and referring to the number and age of the children. These are mutually exclusive variables and include the categories: (1) having children between age 0 and 6 years living in the household (the respondent has children aged 6 or under = 1, all the rest = 0), and (2) children between age 7 and 18 years living in the household^16^ (the respondent has children between 7 and 18 years old = 1, all the rest = 0), no children = 1, all the rest = 0 (the omitted category)^17^.

The immigration status variable will be used as three different sets of categories. The first variable is a dummy variable and will represent the status of immigrant; immigrant = 1; native-born women = 0. The second variable related to immigration will differentiate between main regions of immigrant origin. This variable will be calculated as a series of dummy variables: (1) Immigrants from Sub-Sahara Africa = 1, all the rest = 0. (2) Immigrants from Maghreb = 1, all the rest = 0. (3) Immigrants from European countries = 1, all the rest = 0.

In addition, I will control for the following variables: occupation, education (in years), marital status as a dummy variable indicating whether the respondent is married or not (married = 1, not married = 0), age (in years) and working hours (in hours)^18^.

The control variables will be added on the basis of previous studies that have shown their role in labor market integration of mothers and the size of the motherhood penalty. ***Occupational status*** is likely to affect income directly; numerous explanations have been proposed for the relationship between wages and occupational gender segregation. Supply-side explanations argue that women choose specific occupations because of the particular attributes they provide, compensating for the low earnings they offer. The demand-side explanation, by contrast, argues that women are often “pushed” into low-quality jobs due to discrimination and devaluation (Reskin and Roos, 1990; Findlay et al., 2009). Moreover, literature has established that the gender gap in female occupations will be larger than in more neutral occupations since men are in a much stronger position than women. White-collar occupations are expected to be concentrated in the public sector and to be occupied by more educated employees, while blue-collar occupations are more likely to be in the private sector and populated by less educated employees. These differences affect wages, but also affect other dimensions of employment. Thus, occupations are likely to affect wages of mothers, depending on the work conditions they offer. Furthermore, occupation may be correlated with marital status or the country of origin's level of economic development or gender norms. Following Stier and Yaish (2014), four occupational categories are defined: high white-collar (professional, semiprofessional and managerial occupations); low white-collar (clerical and service occupations); high blue-collar (skilled and semi-skilled occupations); and low blue-collar (primarily unskilled occupations).

***Educational attainment*** is closely related to income, where women with lower educational levels tend to have lower employment opportunities, occupational status, and income. Moreover, education may act as a control for selection into motherhood, as literature has demonstrated the relationship between educational level and fertility delays (Amuedo-Dorantes and Kimmel, 2005; Wilde et al., 2010).

***Marital status*** is also found to be a significant variable affecting the motherhood penalty. Married women may have more financial resources, and thus may be able to choose not to work, or to work in part time jobs, because their partner's income may suffice to support the family's needs. On the other hand, the existence of a partner can enable the couple to share family responsibilities and thus it may be easier for married women to look for a rewarding employment (Budig and England, 2001). Single mothers may need to work longer hours in order to support their children, resulting in higher employment rates and higher income among unmarried women. Conversely, we also observe a reverse tendency, where single mothers may be unable to work since they are the sole caregivers of their children. It should be noted, however, that single mothers receive rent allowance and child day care for all is free in France, enhancing their capacity of employment^19^.

The ***Age*** of an individual is likely to influence work perspectives in different ways. Prior research has demonstrated that discrimination can be made by employers on an age basis. Older people may face difficulties in finding a job or early retirement layoffs, as employers prefer a younger workforce (Rosen and Jerdee, 1977). In addition, since measures of actual experience were not available in the data, age serves as a proxy of potential experience.

Wages and salary are directly influenced by ***Working hours*** and women with children may prefer to be employed as part-time rather than full-time workers to reduce work-family conflict (Stier and Lewin-Epstein, 2003).

Finally, the variable “living in sensitive urban area” will be added to the models when performing the Heckman two-step procedure to account for potential selection bias. ***Sensitive*** ***urban areas*** are dense urban settlements with low-income households and high unemployment rates. They have become increasingly stigmatized, particularly following the urban riots of 2005 (Lagrange, 2006). Scholars have also demonstrated that residents of sensitive urban areas have lower chances of finding a job. Residents of France's most deprived neighborhoods have an increased risk of being unemployed, and address-based discrimination targeting residents of France's peripheral urban areas is prevalent in job and housing markets (Rathelot, 2014; Aeberhardt et al., 2015).

### Methods

In order to examine the hypotheses, the Results chapter will include two main sections. In the first section, I will present descriptive results and characterize immigrant and natives socio-economic and socio-demographic disparity patterns across native women and immigrants in general and by origin groups. The second section will present a multiple linear regression analysis, predicting the effect of motherhood and immigration in addition to the effect of motherhood and region of origin on the wages of women in the French labor market.

Immigrant women might have unobserved characteristics that may cause lower rates of employment, and numerous factors can affect women's decision to seek work. Therefore, in order to account for the selection bias into employment of different groups of women, a two-step Heckman procedure for wage prediction will be performed. In the first step, I used a probit model explaining the selection of women into employment. In the second step, I estimated the wage equation correcting for selection by incorporating a transformation of these predicted individual probabilities as an additional explanatory variable. For the model to be identified, I chose a selection variable that affects the probability of employment, but not wages directly. The standard variables such as marital status, age, years of education were used, as well as including an “***urban sensitive area***” affecting the probability of being employed but not necessarily the level of earnings. The regression models will be performed for native-born women and for each origin group of immigrants separately. The linear model can be represented by the following equation^20^:


$$\begin{array}{l} z=\frac{\beta_{1}+\beta_{2}}{\sqrt{{\left(SE\beta_{1}\right)}^{2}+{\left(SE\beta_{2}\right)}^{2}}} \end{array}$$

Where β1 is the coefficient for natives, β2 is the coefficient for first-generation immigrants, and SEβ1 is the estimated standard error of the coefficient for natives, and SEβ2 is the estimated standard error of the coefficient for immigrants.


(1) $$\begin{array}{r} y=a+b_{i}x_{i} \end{array}$$

where ***y*** is the log of yearly income, and ***x*** is the vector of individual characteristics, including the existence of children by age group, type of occupation, marital status, age, years of education, and weekly working hours. The reason for performing regression analysis for each group of immigrant status (i.e., native, first-generation immigrants) separately is multipurpose; first, it allows us to understand not only the interaction between immigration status and motherhood, but also the interaction between immigration status and the other control variables, such as marriage and education. Moreover, it also enables us to distinguish between the cost of the region of origin and the cost of immigration. Finally, estimating the motherhood effect for each immigrant group separately allows us to account for some of the heterogeneous characteristics across the different groups of women.

To illustrate the results, different profiles of women will be displayed by immigration status and motherhood. The income of women in the French labor market, will be calculated for each group with and without children for a 37 year old woman^21^, working 35 h weekly^22^, in a low white-collar type of occupation^23^ with 10^24^ years of education, married, with children under 18 (7–18 years old) and under 6 (0–6 years old).

## Chapter 3: Analysis and Findings

### Descriptive Overview

This section introduces the descriptive socio-demographic and socio-economic characteristics of the analyzed sample comparing native French women and immigrant women (Table 1) and across origin groups (Table 2). Since second-generation immigrant women were born in France and have different socio-demographic and socio-economic characteristics than the first-generation population, I chose to compare the first generation of immigrants to the entire native-born population^25^.

**Table 1:** Descriptive statistics by immigration status (aged 18–50).

	**All population (mothers and childless, employed and not employed)**		**Employed mothers only**	
	**Native-born**	**Immigrants**	**Native-born**	**Immigrants**
Children 7–18%	34%	32%	60%	59%
children under 6%	26%	37%	40%	41%
Employed%	78%	54%		
Yearly wages (only for employed women)	€18,772	€16,808	€19,196	€16,313
(std deviation)	(10,827)	(11, 741)	(11,125)	(11,797)
Low Blue collar	4.6%	10.9%	4.2%	11.4%
High Blue collar	6.6%	5.4%	5.7%	4.8%
Low White collar	74.7%	70.2%	75.8%	71.8%
High White collar	14.1%	13.6%	14.2%	11.8%
Married %	44%	65%	58%	69%
Years of education	11.36	9.22	11.99	10
(std deviation)	(3.91)	(5.2)	(3.53)	(5.09)
Age	36.49	37.57	38.25	39.13
(std deviation)	(8.87)	(8.10)	(6.82)	(6.91)
Number of children	1.78	2.01	1.70	1.79
(std deviation)	(1.01)	(1.26)	(0.74)	(0.86)
Weekly working hours	34.56	32.9	33.84	32.1
(std deviation)	(9.54)	(11.89)	(9.24)	(10.69)
Years since immigration		18.97		
(std deviation)		(12.73)		
FR nationality%	100%	43%	100%	56%
*N*	73,180	11,896	29,675	3,505

*SOURCE: Enquête sur les revenus fiscaux et sociaux (ERFS), INSEE 2009–2012*.

**Table 2:** Descriptive statistics by immigration status and region of origin (age 18–50).

	**All population (mothers and childless, employed and not employed)**			**Employed mothers only**		
	**Immigr europe**	**Immigr magrheb**	**Immigr sub-Sahara**	**Immigr europe**	**Immigr magrheb**	**Immigr sub-Sahara**
Children 7–18%	36%	31%	30%	65%	60%	50%
children under 6%	26%	43%	43%	35%	40%	50%
Employed%	68%	43%	58%			
Yearly wages (only for employed women)	€18,870	€16,044	€15,245	€18,686	€15,464	€14,732
(std deviation)	(13.220)	(11,135)	(9,493)	(13,788)	(11,307)	(9,009)
Low Blue collar	8.2%	12.5%	11.6%	8.7%	11.7%	12.7%
High Blue collar	5.8%	4.0%	3.5%	4.9%	3.6%	3.3%
Low White collar	69.5%	70.8%	77.3%	71.2%	72.8%	77.7%
High White collar	16.5%	12.6%	7.5%	15.1%	11.8%	6.2%
Married %	62%	72%	47%	70%	73%	54%
Years of education	10.41	8.45	8.93	10.56	10	9.33
(std deviation)	(5.03)	(5.11)	(4.93)	(5.01)	(5.01)	(4.87)
Age	38.56	37.76	36.63	39.62	39.88	37.95
(std deviation)	(8.14)	(8.36)	(7.86)	(6.70)	(7.21)	(7.04)
Number of children	1.74	2.16	2.17	1.60	1.88	1.97
(std deviation)	(0.82)	(1.06)	(1.39)	(0.71)	(0.88)	(1.01)
Weekly working hours	33.28	32.59	32.21	32.13	32.24	31.73
(std deviation)	(10.76)	(10.74)	(9.69)	(10.97)	(11.27)	(9.45)
Years since immigration	20.76	20.87	17.63	23.71	25.55	19.89
(std deviation)	(14.36)	(1379)	(10.77)	(13.81)	(12.68)	(10.56)
FR nationality%	36%	52%	47%	56%	72%	58%
*N*	3,619	4,024	2,252	1,229	998	809

*SOURCE: Enquête sur les revenus fiscaux et sociaux (ERFS), INSEE 2009–2012*.

The descriptive overview (Table 1) presents the socio-economic characteristics of (1) native-born French women and (2) all foreign-born women pooled together, as well as an additional panel for employed mothers only, for each group of women. The results suggest that the rate of women with children in the household is higher among the immigrant population than the rate of women with children among the native born. The rate of women with children between 7 and 18 years old is quite similar for both groups, with a slightly higher percentage of children between 7 and 18 years old among native born women (34 vs. 32%). The rate of children under 6 years old is the highest among the immigrant population (37 vs. 26%). Among employed mothers, both native-born women and immigrants have a similar rate of children under 6 years old and under 18 years old (around 60% of children between 7 and 18 years old and 40% of children under 6 years old).

The employment rate is higher among native French born women (78%) than among the foreign born population (54%). The average income is calculated for employed women only (women working more than 5 h weekly). Native-born women earn on the average 2000€ more than first-generation immigrant women. As for employed mothers, native-born women earn as much as 3000€ more than employed immigrant mothers. Most women work in low white-collar types of occupations and the rate of women working in low white, high blue and high white-collar occupations are quite similar for both immigrants and native-born populations. It should be noted, however, that many more immigrants work in low blue-collar type of occupations compared to the native-born population.

Table 2 presents the socio-economic and socio-demographic characteristics of women aged 18–50 by region of origin and immigration status. The table is divided into 3 groups: (1) first-generation immigrant women from Europe, (2) first-generation immigrant women from Maghreb, and (3) first-generation immigrant women from sub-Saharan Africa.

Table 2 presents the socio-economic characteristics of foreign-born women for each origin group, as well as an additional panel of employed mothers only for each group of women. The results in Table 2 suggest that immigrants from Maghreb and sub-Sahara have the highest rate of women with children (30% + 43% = 73% for immigrants from Sub Sahara and 74% for immigrants from the Maghreb) and in particular have the highest rate of children under 6 years old (43%).

The primary set of results are consistent with the literature suggesting that immigrant women from developed countries of origin have better labor market outcomes than immigrants from poor countries. Immigrant women from Europe are the group with the highest employment rate after native women. The descriptive results support the assumptions regarding the level of traditionalism with regard to gender roles in the country of origin, and immigrants from the Maghreb seem to suffer from the lowest employment rates (43%). Although gender ideology prevailing in sub-Saharan countries promotes economic participation of women, immigrants from sub-Sahara seem to do worse in terms of employment rates than immigrants from Europe; however, they appear to do much better compared to immigrants from Maghreb. The average annual wage is calculated for employed women only. Noticeably, the highest average income is among immigrant women from Europe (€18,772). Once more and as expected, immigrant women from Europe seem to be positively selected in the French labor market as they benefit from the highest income compared to immigrants from other regions of origin. The group with the lowest average annual income is the first-generation of immigrant women from sub-Sahara (€15,245)^26^. Among employed mothers, the income of immigrants from Europe is still the highest and is similar to the income of employed mothers in general. however, the income of employed mothers from Maghreb and sub-Sahara seems lower among employed mothers compared to employed women from the same origin group. The highest rate of high white-collar are among immigrants from Europe and the lowest among immigrants from sub-Sahara. In addition, both immigrants from Sub-Sahara and immigrants from Maghreb seem to be overrepresented in low blue-collar type of occupation with little difference between employed mothers and employed women in general, suggesting low selectivity of these women into the French labor market.

## Regression Analysis

### The Consequence of Immigration Status and Motherhood Status on Wages

In this section I examine the effect of motherhood and region of origin on the annual income of women in the French labor market.

In order to estimate whether and to what extent motherhood status and immigration status affect the yearly income of women in the French labor market, I will present below two models of linear regression for each group of women separately according to their immigrant status. I will also present two models correcting for selection bias according to the two-step Heckman selection model (1979), in which the first generation will be compared to the native-born population.

Columns (2)–(4) are analogous to Columns (1)–(3) but correct for selection bias. The results in Column 1 show that the yearly income of native-born women with children under 18 years old is lower than the yearly income of native-born women without children, since the negative effect of children under 18 years old is statistically significant. Native-born women with children between 7 and 18 years old earn 4.5% less than women without children. Contrary to expectation, having children under 6 has a positive effect on wages for native-born women. Native-born women with children under 6 years old earn 4.9% more than women without children.

The results for the two-step Heckman model reveal that when correcting for selectivity, the negative effect of motherhood among native-born women (Column 2) for children under 18 years stays the same and the positive effect of young children on wages of native-born women does not change much either (4.5% instead of 4.9%). The Lambda term is statistically not significant. The results in Column 3 show that the effect of children under 18 years old on immigrants is not statistically significant. Moreover, contrary to native women, the effect of children under 6 years old on immigrant women is negative. First-generation immigrant women with children under 6 years old earn 6.9% less than immigrant women without children. Correcting for selectivity for the immigrant population (Column 4) renders the coefficient of children under 18 years old much more similar to that of the native-born population. The negative effect of children under 18 years old becomes statistically significant (5.6% lower yearly wages) and the effect of children under 6 years old becomes statistically not significant. In contrast with the native-born population, the Lambda term is statistically significant and negative, meaning that among the immigrant women, the population with the highest probability to work will actually be the one likely to earn the least.

In order to test the hypothesis and to understand if there is a differentiated motherhood effect on immigrant women compared to native women, the statistical significance between the effect of children on native-born women and the effect of children on first-generation immigrant women is tested. The statistical significance test performed on the uncorrected models reveals that the difference between the native-born women and first-generation immigrants in the motherhood penalty for children under 18 years old, is not statistically significant. However, the difference between the effect of children under 6 years old on first-generation immigrants and such effect on native-born women is statistically significant^27^. The corrected significance test between the effect of children for the native-born and the first-generation immigrant population demonstrates, however, that the difference between them is not statistically significant for having children under 6 and for children between 7 and 18 years old^28^.

Nevertheless, the premium observed for children under 6 years old among native women is counterintuitive, as one would expect that the motherhood penalty should be higher for young children that need extensive amounts of care, which is difficult to reconcile with high paying occupations. Moreover, if there would be a premium for motherhood in France, it would have been logical to observe it among mothers with older children, since scholars have often related the motherhood penalty to child rearing, caregiving, and family responsibility necessary primarily for younger age children (Anderson et al., 2003; Amuedo-Dorantes and Kimmel, 2005). However, it seems that the penalty observed in France for young children is not pronounced in terms of wages. A possible explanation for the motherhood premium for children under 6 years old could be the result of free daycare in France and family-friendly policies. Children have access to publicly subsidized home-based care, accredited family daycare providers, and nursery from a very young age (Lucifora et al., 2017). Moreover, motherhood penalty literature in the cross-national perspective has often revealed a smaller motherhood penalty in France and Belgium as well as in southern European countries.

Consistent with previous studies on the motherhood penalty for native women in France, the motherhood penalty is small (Budig et al., 2016; Cukrowska-Torzewska and Lovasz, 2020)^29^. The results on the French labor market show a mechanism where a penalty in terms of wages for children under 18 years old is observed. These results indicate a pattern of a long-term disadvantage as opposed to an immediate penalty. In fact, the penalty observed for women with older children might be as a result of a cumulative loss in human capital due to career interruption.

In order to display the results concretely and to understand the relative motherhood effect on immigrant women, the yearly wages of a woman in the French labor market are calculated for the native-born population and the first-generation immigrant population. The graph is based on Table 3, Model 1 and 3 (uncorrected for selection) and Model 2 and 4 (corrected for selection models), with and without children. The yearly wages are calculated for a 37-year-old woman, married, with or without children under 18 years old and with 10 years of education for each group separately. The graphs are presented for a woman with children under 18 years old, as the results above have demonstrated that the negative effect of children on women's wages are pronounced among women with children under 18. Moreover, taking into account children under 18 years old allows us to portray longer time effects of motherhood on wages.

**Table 3:** Regressions predicting the Logarithmic transformation of the yearly wages (LN income) of women in the French labor market (18–50 years old) according to their immigration status, and Heckman selection model correcting for selectivity.

	**Natives**		**1st GEN Immigrant (foreign born)**		**Significance test between the models (uncorrected)**	**Significance test between the models (corrected)**
	**Uncorrected for selectivity**	**Corrected for selectivity**	**Uncorrected for selectivity**	**Corrected for selectivity**	**Natives and immigrants**	**Natives and Immigrants**
	(1)	(2)	(3)	(4)		
Children under 18	−0.045^***^	−0.045^***^	−0.009	−0.056^*^	Not significant	Not significant
	(0.01)	(0.01)	(0.02)	(0.03)		
Children under 6	0.049^***^	0.045^***^	−0.069^*^	0.040	^***^	Not significant
	(0.01)	(0.01)	(0.03)	(0.04)		
LowBlue collar	−0.056^***^	−0.048^**^	0.002	−0.252^***^	Not significant	^***^
	(0.01)	(0.02)	(0.03)	(0.03)		
HighBlue collar	0.143^***^	0.156^***^	0.112^*^	−0.139^*^	Not significant	^***^
	(0.01)	(0.02)	(0.04)	(0.08)		
HighWhite collar	0.407^***^	0.414^***^	0.561^***^	0.349^***^	^***^	Not significant
	(0.01)	(0.01)	(0.03)	(0.06)		
Married	−0.066^***^	−0.065^***^	−0.059^**^	0.007	Not significant	^***^
	(0.01)	(0.01)	(0.02)	(0.02)		
Age	0.020^***^	0.021^***^	0.014^***^	0.007^***^	^***^	^***^
	(0.00)	(0.00)	(0.00)	(0.00)		
Educ years	0.050^***^	0.052^***^	0.022^***^	0.003	^***^	^***^
	(0.00)	(0.00)	(0.00)	(0.01)		
Weekly working hours	0.020^***^	0.020^***^	0.028^***^	0.027^***^	^***^	^***^
	(0.00)	(0.00)	(0.00)	(0.00)		
Constant	7.552^***^	7.473^***^	7.736^***^	8.673^***^	Not significant	^***^
	(0.02)	(0.11)	(0.07)	(0.25)		
Lambda		0.053		−0.585^***^		^***^
		(0.06)		(0.15)		
R-squared	0.275		0.293			
N cases	49,673	46,966	5,350	5,319		

* *p < 0.1*;

** *p < 0.05*;

*** *p < 0.01*.

*SOURCE: Enquête sur les revenus fiscaux et sociaux (ERFS), INSEE 2009–2012*.

**CHART 1:**
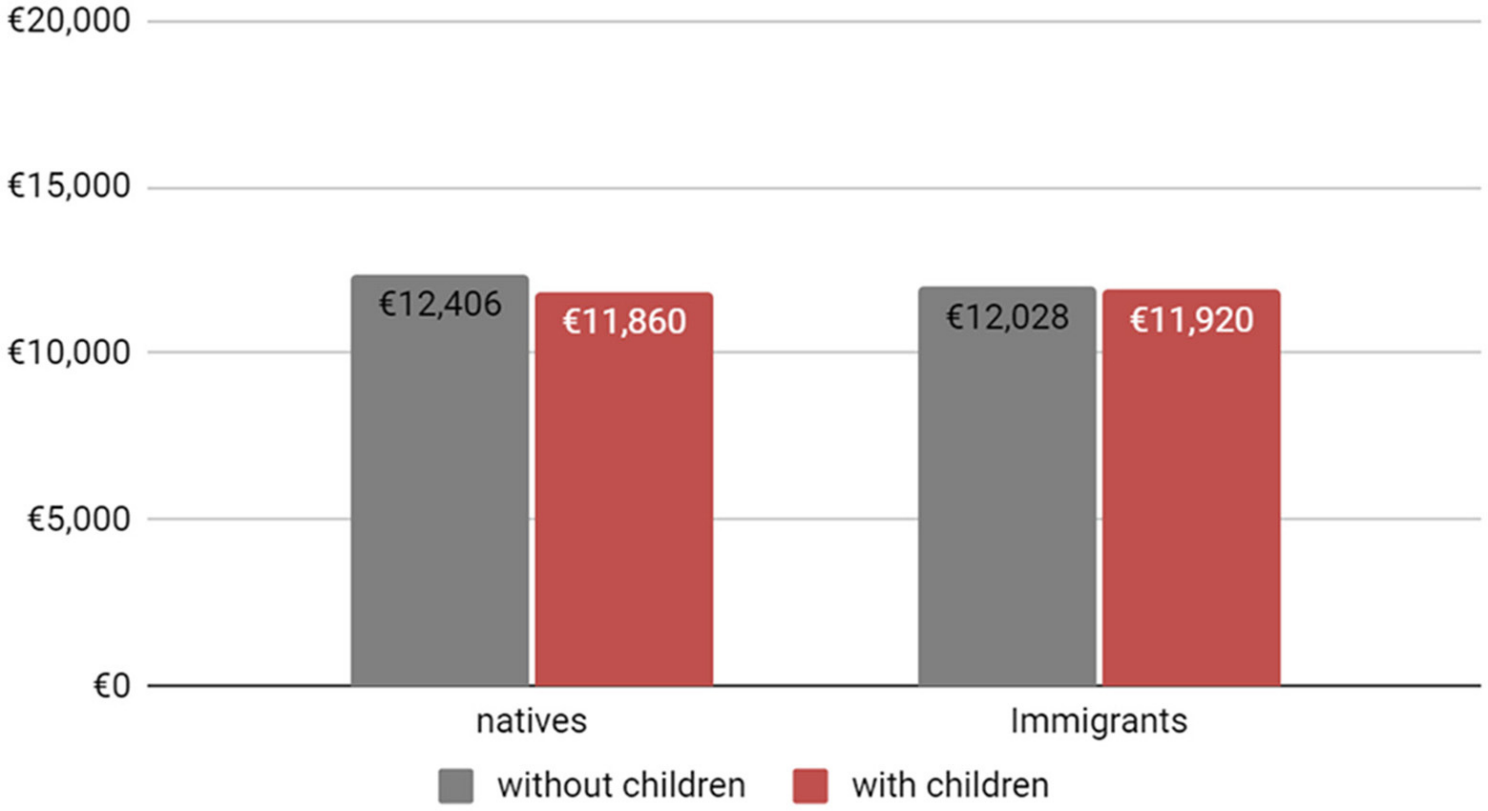
Yearly income of women in the French labor market by immigration status and children **uncorrected** for selection (under 18 years old). SOURCE: Enquête sur les revenus fiscaux et sociaux (ERFS), INSEE 2009–2012.

**CHART 2:**
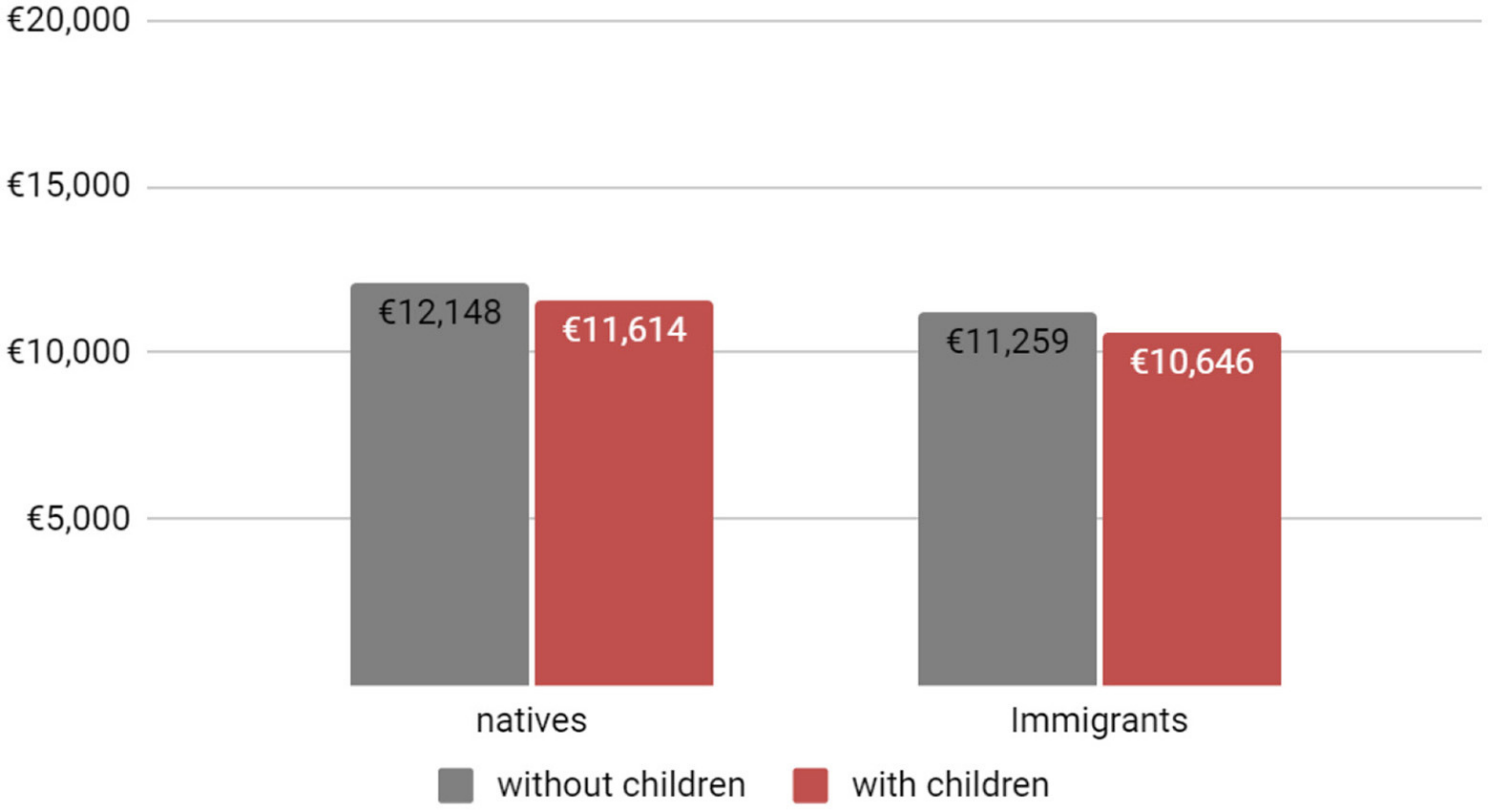
Yearly income of women in the French labor market by immigration status and children **corrected** for selection (under 18 years old). SOURCE: Enquête sur les revenus fiscaux et sociaux (ERFS), INSEE 2009–2012.

As presented in Chart 1 and based on the models uncorrected for selection, only native-born women with children under 18 years old suffer from a motherhood penalty in terms of yearly wages. Yet, contrary to expectation based on the uncorrected model, immigrants bear no motherhood penalty for children between 7 and 18 years old. However, based on the models corrected for selection (Chart 2), we can observe that both a native-born and an immigrant woman with children under 18 years old will suffer from a motherhood penalty in terms of yearly wages and the motherhood penalty of immigrant women becomes greater compared to native women. In addition, after controlling for selection, the income of immigrant woman becomes much lower as compared to the uncorrected model.

In summary, based on the results above, controlling for selection widens the pay gap between native and immigrant women in general. These results assert the existence of a selection bias into employment, yet the selection mechanism for the immigrant population is negative for the immigrant population resulting in the yearly wages of immigrants in being even lower than without correcting for selection.

### The Consequence of Region of Origin and Motherhood Status on Wages

To examine the effect of motherhood and the effect of country of origin, three models of linear regression will be performed (Table 4) for the three immigrant groups coming from different regions of origin, and the native-born population separately, as well as three models correcting for selection bias according to the two-step Heckman selection model (*1979*) for the three immigrant groups coming from different regions of origin, and for the native-born population separately^30^. In order to understand whether there is a differentiated motherhood effect on immigrant women from different countries of origin compared to native women, the significance between the effect of children on native women and the effect of children on each immigrant group of immigrant women will be tested for both the corrected and uncorrected models.

**Table 4:** Linear Regression predicting the logarithmic transformation of the yearly income of women in the French labor market according to the region of origin and motherhood status (18–50 years).

	**Natives**		**1st Gen Europe**		**1st Gen Maghreb**		**1st Gen Sub-Sahara**	
	**Uncorrected for selectivity**	**Corrected for selectivity**	**Uncorrected for selectivity**	**Corrected for selectivity**	**Uncorrected for selectivity**	**Corrected for selectivity**	**Uncorrected for selectivity**	**Corrected for selectivity**
	(1)	(2)	(3)	(4)	(5)	(6)	(7)	(8)
Children under 18	−0.045^***^	−0.045^***^	0.037	−0.002	−0.109^**^	−0.127^**^	−0.014	−0.140
	(0.01)	(0.01)	(0.03)	(0.05)	(0.05)	(0.05)	(0.06)	(0.12)
CHILDREN under 6	0.049^***^	0.045^***^	0.086^**^	0.196^**^	−0.180^***^	−0.059	−0.138^**^	0.079
	(0.01)	(0.01)	(0.04)	(0.08)	(0.06)	(0.17)	(0.06)	(0.08)
LowBlue collar	−0.056^***^	−0.048^**^	−0.017	−0.172	−0.009	−0.264	0.052	−0.289
	(0.01)	(0.02)	(0.05)	(0.11)	(0.06)	(0.20)	(0.07)	(0.22)
HighBlue collar	0.143^***^	0.156^***^	0.189^**^	−0.018	0.121	−0.052	0.049	−0.290
	(0.01)	(0.02)	(0.06)	(0.14)	(0.10)	(0.17)	(0.11)	(0.26)
HighWhite collar	0.407^***^	0.414^***^	0.551^***^	0.309^**^	0.565^**^	0.421^***^	0.485^***^	0.172
	(0.01)	(0.01)	(0.05)	(0.14)	(0.07)	(0.13)	(0.09)	(0.22)
Married	−0.066^***^	−0.065^***^	−0.044	0.002	−0.096^**^	−0.026	−0.044	−0.101
	(0.01)	(0.01)	(0.03)	(0.05)	(0.05)	(0.07)	(0.05)	(0.10)
Age	0.020^***^	0.021^***^	0.013^***^	0.007	0.017^***^	0.011^*^	0.012^***^	−0.004
	(0.00)	(0.00)	(0.00)	(0.01)	(0.00)	(0.01)	(0.00)	(0.02)
Educ years	0.050^***^	0.052^***^	0.019^***^	0.010	0.027^***^	0.006	0.020^***^	−0.010
	(0.00)	(0.00)	(0.00)	(0.01)	(0.00)	(0.02)	(0.01)	(0.02)
Weekly working hours	0.020^***^	0.020^***^	0.026^***^	0.026^***^	0.025^***^	0.025^***^	0.034^***^	0.034^***^
	(0.00)	(0.00)	(0.00)	(0.00)	(0.00)	(0.00)	(0.00)	(0.00)
Constant	7.552^***^	7.473^***^	7.862^***^	8.679^***^	7.699^***^	8.513^***^	7.661^***^	9.581^***^
	(0.02)	(0.11)	(0.10)	(0.45)	(0.14)	(0.70)	(0.16)	(1.13)
Lambda		0.053		−0.693		−0.432		−1.158
		(0.06)		(0.37)		(0.31)		(0.67)
R-squared	0.275		0.320		0.272		0.255	
N cases	49,673	46,966	2,050	1,982	1,498	1,463	1,101	1,062
	**Significance between models (Uncorrected Models)**				**Significance between models (Corrected Models)**			
	**Natives and 1st gen Europe**	**Natives and 1st gen Maghreb**		**Natives and 1st gen sub-Sahara**	**Natives and 1st gen Europe**	**Natives and 1st gen Maghreb**		**Natives and 1st gen sub-Sahara**
Children under 18	^***^	Not significant		Not significant	Not significant	Not significant		Not significant
Children under 6	Not significant	^***^		^***^	Not significant	Not significant		Not significant
LowBlue colar	Not significant	^***^		Not significant	Not significant	Not significant		Not significant
HighBlue colar	Not significant	Not significant		Not significant	Not significant	Not significant		Not significant
HighWhite colar	^***^	^***^		Not significant	Not significant	Not significant		Not significant
Married	Not significant	Not significant		Not significant	Not significant	Not significant		Not significant
Age	^***^	^***^		^***^	Not significant	Not significant		Not significant
Educ years	^***^	^***^		^***^	^***^	^***^		^***^
Weekly working hours	^***^	^***^		^***^	^***^	^***^		^***^
Constant	^***^	Not significant		Not significant	^***^	Not significant		Not significant
Lambda				^***^	Not significant	Not significant

* *p < 0.1*;

** *p < 0.05*;

*** *p < 0.01*.

*Source: Enquête sur les revenus fiscaux et sociaux (ERFS), INSEE 2009–2012*.

Columns (2)–(4), (6), and (8) are analogous to Columns (1)–(3), (5), and (7) but correct for the selection bias.

The results of Model 3 (Column 3; Table 4) suggest that there is no motherhood penalty in terms of wages on having a child under 18 years for first-generation immigrant women from Europe, both in the corrected and uncorrected models. Noticeably, the coefficient on children under 6 years is positive for first-generation immigrant women from Europe; that is, there is a motherhood premium on having children under 6 years old. Having children under 6 years adds 8.6% to the annual income compared to first-generation immigrants from Europe without children.

Model 5 (Column 5), indicates that there is a motherhood penalty in terms of wages for immigrants from Maghreb both for children between 7 and 18 years old and for children under 6 years old. Immigrants with children under 18 years from Maghreb earn 10.9% less than childless immigrants from Maghreb. The negative effect of having children under 6 years old on the annual income of immigrant women from Maghreb is even stronger and immigrant mothers with young children earn as much as 18% less than childless immigrant women from Maghreb.

The results of Model 7 (Column 4) indicate that the coefficient for children between 7 and 18 years old is not statistically significant for immigrant women from sub-Sahara. However, the effect of children under 6 years old is negative and significant. Having children under 6 years old for immigrant women from sub-Sahara lowers the annual wages by 13.8%. Notably, the Lambda terms are not statistically significant in the corrected selection models across all groups^31^.

In order to understand whether there is a differentiated motherhood effect on immigrant women compared to native women, the significance of the difference between the effect of children on native women and the effect of children on immigrant women from different regions of origin is tested. The significance test demonstrates that the differences in the effect of children under 18 years old on first-generation immigrants from Europe and native-born women are statistically significant, while the differences between the effect of children under 18 years for natives and immigrants from Maghreb and sub-Saharan Africa are not statistically significant.

The difference in the effect of child under 6 years old on first-generation immigrants is statistically significant between native-born women and first-generation immigrant women from both Maghreb and sub-Saharan Africa. Immigrant women from sub-Sahara and from Maghreb suffer from a significant wage penalty compared to native mothers of children under 6 years^32^.

Although, immigrant women from Maghreb seem to have the highest return on education compared to every other immigrant group, the results of the effect of children on the yearly wages show evidence of them being particularly disadvantaged by motherhood. Consistently with previous studies and research hypotheses, women immigrating from Maghreb, bears additional penalty on being married, and each hour worked rewards them less than any other group. Women from Maghreb suffer from a motherhood penalty on both children under 18 years old and children under 6 years old.

First-generation women from Sub-Sahara are also a disadvantaged group. They have the lowest benefit of age and the lowest return on education from all groups. These results suggest that their wages are dependent primarily on the hours worked and less on education and experience; typically, the lower paying types of jobs. Moreover, they suffer from a significant motherhood penalty for children under 6 years old.

Finally, and consistent with the hypothesis of this research, the least disadvantaged group in terms of motherhood penalty are immigrants from Europe, who have a statistically significant motherhood premium for younger children and bear no penalty for children under 18 years old. The effect of marriage on yearly wages is not significant and their return on education is better than immigrant from Sub-Sahara.

**CHART 3:**
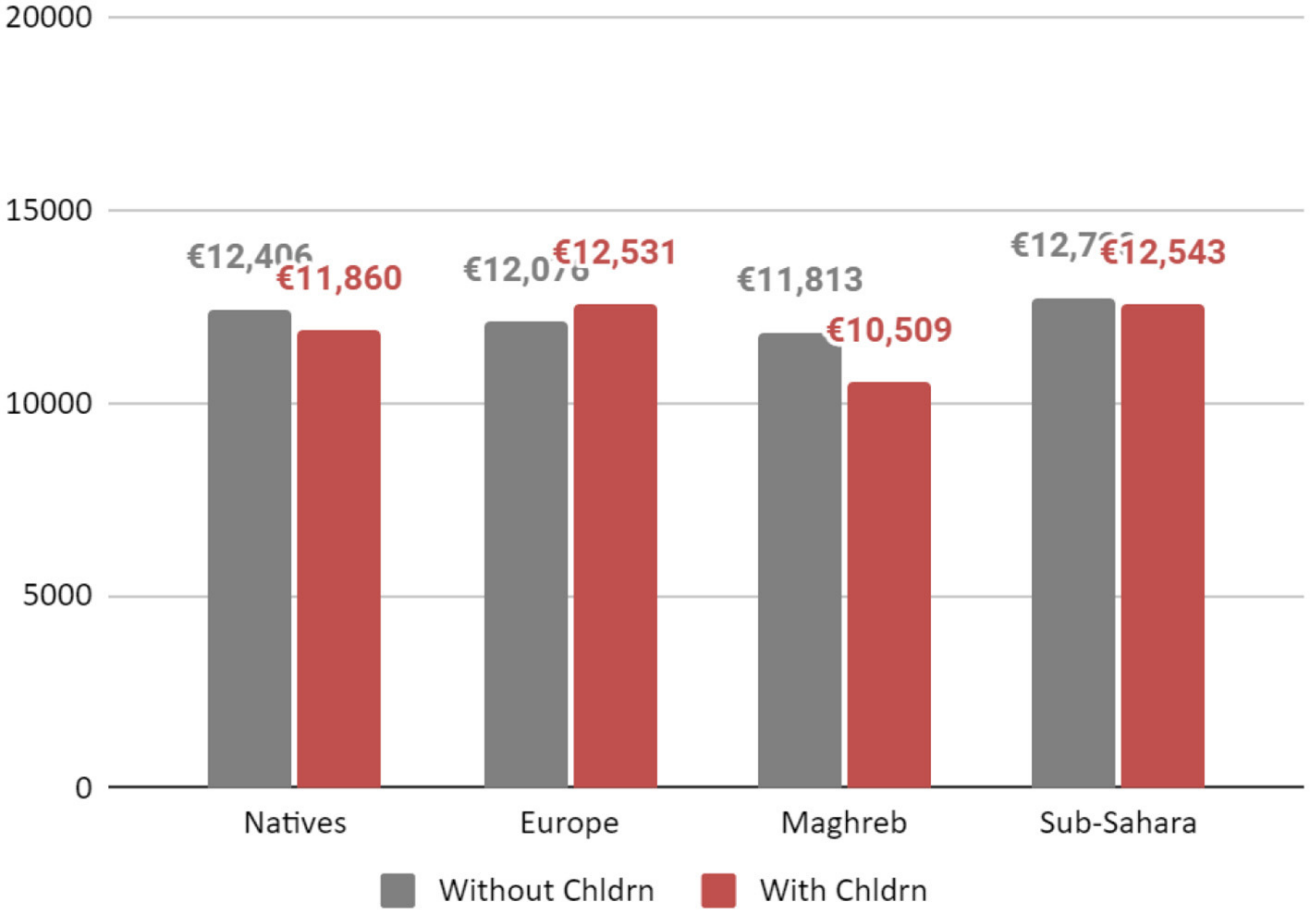
Yearly income of a woman in the French labor market by immigration status and children (under 18 years old). Source: Enquête sur les revenus fiscaux et sociaux (ERFS), INSEE 2009–2012.

**CHART 4:**
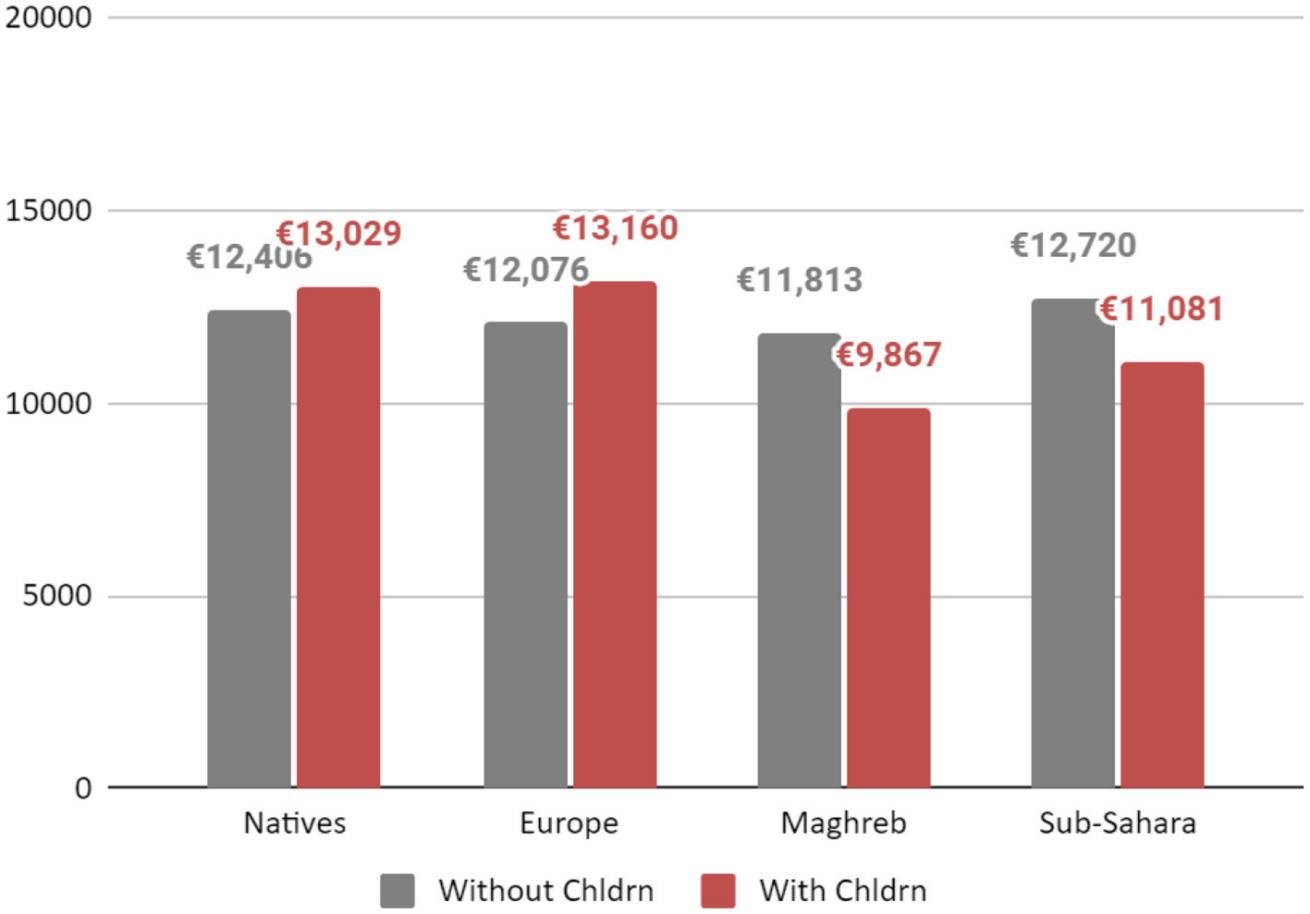
The yearly income of a woman, in the French labor market according to origin groups and children under 6 years old. Source: Enquête sur les revenus fiscaux et sociaux (ERFS), INSEE 2009–2012.

In order to display the results concretely, Chart 3 demonstrates the differences between each group in terms of their relative motherhood penalty for having children between 7 and 18 years old and Chart 4 presents the differences in the relative motherhood cost for each group for having children under 6 years old. The annual wages of a woman in the French labor market are based on Table 2, Models 1, 3, 5, and 7^33^. The wages are calculated for a 37-year-old woman, working in a low white-collar occupation, married, with or without children (under 6 and between 7 and 18 years old) and with 10 years of education for each group separately.

As shown in Chart 3, and contrary to every other group, an immigrant woman from Europe with children under 18 experiences no motherhood penalty in the French labor market. However, a motherhood premium cannot be asserted since the coefficient for having children between 7 and 18 for immigrants from Europe was not statistically significant. An immigrant woman from sub-Sahara with children under 18 earns nearly the same wages as an immigrant woman from sub-Sahara without children. An immigrant from Maghreb will be the woman suffering the most significant motherhood penalty for having children between 7 and 18 years. As expected, and as seen in previous results, a woman from Maghreb will also earns less than all other groups even if childless.

As it appears in Chart 4, both a native woman and immigrant woman from Europe with children below age 6, will experience a motherhood premium although the premium is much greater for immigrants from Europe. By contrast, both immigrants from the Maghreb and sub-Sahara will have a motherhood penalty for children under 6 years old. The most significant motherhood penalty for children under 6 years old, however, will be for an immigrant from the Maghreb.

## Discussion

Give the absence of research focusing on the interactive effect of immigration and motherhood on women's earnings, this study seeks to contribute to the body of literature on gender and immigration by analyzing the interactive effect of motherhood, immigration, and region of origin on women's labor market integration in France. Although prior research has identified numerous factors shaping the motherhood penalty at both the individual and the contextual level, to the best of my knowledge no earlier study has examined the relative motherhood cost for immigrant women, as compared to native-born women, with reference to the origin group of the immigrant.

It is well-established in the literature that the cost of immigration varies among different immigrant groups. Factors such as the cultural similarity of the country of origin with the culture of the host country, the level of economic development of the country of origin and religious similarity can shape the selection mechanism of immigrants (Stier and Tienda, 1992; Raijman and Semyonov, 1997; Hondagneu-Sotelo, 2003; Inglehart and Norris, 2003; Diehl et al., 2009). When evaluating the economic integration of immigrant women, however, the literature often fails to consider the difference between immigrant women with children and those without children, the cost of motherhood for the immigrant, and how the region of origin of these women may affect the cost of motherhood.

This study focused on immigrant women from three major regions of origin—Europe, the Maghreb, and sub-Saharan Africa. Specifically, I attempted to perform the following analysis (1) An examination of whether immigration status and motherhood affect women's wages and to what extent, (b) An examination of the effect of the motherhood penalty on the wages of immigrant women compared to native women (interactive effect) (c) An analysis of the effect of motherhood on women's wages across immigrant groups from different regions of origin.

Using the “Enquête des Revenus Fiscaux et Sociaux,” 2009–2012 (INSEE, 2009, 2010, 2011, 2012), the results of this study confirmed that the motherhood disadvantage is not uniform across the different origin groups of immigrant women. The results support the hypothesis regarding a lower wage for immigrant mothers as compared to childless immigrants. In addition, the results are consistent with the hypothesis regarding a greater motherhood penalty for immigrants in general for children under age 6. Moreover, the results supported the hypothesis regarding an interactive cost between the region of origin and motherhood for immigrant women. The findings assert the existence of a greater motherhood penalty which is especially pronounced for immigrant mothers coming from less economically developed countries and immigrant mothers coming from traditional countries with regard to gender roles (mothers from the Maghreb and sub-Saharan Africa). Indeed, immigrants from the Maghreb experienced the worst motherhood penalty in terms of income; they had a motherhood penalty both for having children under 18 years old and for children under 6 years old. Immigrants from sub-Sahara also experienced a significant motherhood penalty for children under 6 years old; however, they did not suffer from a motherhood penalty for older children (7–18 years old). Immigrants from Europe had no motherhood penalty—not for having children between 7 and 18 years old, and they even had a premium for having children under 6 years old. It seems that in accordance with the research hypothesis, immigrants from Europe did better than immigrants from the Maghreb and sub-Sahara. They did even better than native mothers, since while they had no penalty whatsoever, native mothers by comparison had a small motherhood penalty for children under 18 years old (7–18).

Contrary to the working assumption, young children had a positive impact on wages for native women and immigrants from Europe. Not only did these results highlight the importance of considering children's age when investigating the motherhood penalty, they are also extremely interesting as they may suggest a different approach to employment for mothers depending on the age of their children. While children often have negative long-term consequences on wages, these results could be revealing a particularly positively selected group of women with specific attributes, observed or not observed, that are necessary to overcome barriers existing for mothers, especially immigrant mothers of young children.

Although this study has evaluated the interactive effect between immigration status and motherhood status across origin groups, an important limitation needs to be taken into account when interpreting the findings. First, it may be problematic to rely on cross-sectional data for measuring both the motherhood penalty and the economic integration of immigrant women. Indeed, without using longitudinal data, it is impossible to assert a stronger negative effect of motherhood as a result of immigration. However, the rationale behind this study was first and foremost to provide evidence of the importance of considering intersectionality when studying gender, especially when related to immigration. As such, this research establishes that motherhood, immigration, and origin intersect one another, resulting in a combined impact, exceeding their independent and cumulative effects.

Moreover, the effect of the employment patterns of partners were not taking into account in the analysis and thus prevented me from examining its effect on women's employment outcomes. Moreover, partner employment patterns could affect immigrant women especially in the event of tied migration. Unfortunately, these parameters were not provided in the data.

The findings presented in this study contribute to the literature on motherhood and immigration by providing evidence on the intersection of motherhood, immigration, and region of origin in shaping inequality in the labor market. Consequently, this study is not limited to the ways in which motherhood interacts with immigration—it also suggests how mechanisms such as discrimination of immigrants and mothers by employers, lack of transferability of human capital, the effects of partners employment pattern, but also cultural values and social norms related to gender roles, may possibly influence immigrant labor market outcomes in the host country. Moreover, by choosing France, a principal destination of immigration in Europe and where gender, culture, tradition, and the integration of immigrant subgroups is a constant feature of public and academic debates, this study provides a new perspective on the role of motherhood, when intersected with countries of origin in the immigrant integration process.

This research further contributes to the immigration and motherhood literature by suggesting that motherhood could be one of the main mechanisms shaping the disadvantage in the labor market for immigrant women compared to immigrant men. Indeed, while women can be seen as primary movers if neither married nor possessing family responsibilities—under such circumstances their individual wellbeing and success is their main motivation for immigration. By contrast, women with children are more likely to be tied movers. Working on the assumption that the gender gap is likely to be more pronounced for tied migrants, and that women with children are more likely to be tied movers, this study suggests that motherhood could be, as a matter of fact, one of the main mechanisms shaping gender inequality for immigrants in contrast to French natives. Such a finding could motivate further research on the specific motherhood penalty experienced by immigrants, occurring as a result of their immigration experience, as a result of their disadvantaged status or as a result of their distinct cultural attributes.

## Data Availability Statement

The original contributions presented in the study are included in the article/Supplementary Materials, further inquiries can be directed to the corresponding author.

## Author Contributions

The author confirms being the sole contributor of this work and has approved it for publication.

## Funding

Bar-Ilan University provided a 1500NIS (about 450*$*) budget for editing purpose.

## Conflict of Interest

The author declares that the research was conducted in the absence of any commercial or financial relationships that could be construed as a potential conflict of interest.

## Publisher's Note

All claims expressed in this article are solely those of the authors and do not necessarily represent those of their affiliated organizations, or those of the publisher, the editors and the reviewers. Any product that may be evaluated in this article, or claim that may be made by its manufacturer, is not guaranteed or endorsed by the publisher.

## Footnotes

^2^The term “childless” will be used in this study for women without children, whether or not by choice.

^3^Some researchers have established differences between married immigrant women and unmarried immigrant women (Duleep and Sanders, 1993; Baker and Benjamin, 1997) or have used children as a control variable when analyzing the disadvantage experienced by women immigrants. But to the best of my knowledge, no research to date has considered the relative labor market cost of motherhood for immigrants compared to natives.

^4^The term origin groups or region of origin will be used interchangeably throughout this study to describe the different regions of origin of immigrant groups.

^5^The data used in this study lacked country-specific detailed variables, given that the country of birth of the respondent was pooled into five regions only: France, Europe, the Maghreb, sub-Saharan Africa, and other. Moreover, France has a large population of immigrants from Turkey and Asia, not taken into account within this analysis as they were all pooled under the “other” category in the country of origin variable. It should be noted that there are large cultural and socio-economic variations across the diverse countries that make up each region of origin. The socio-economic and cultural differences across the various countries in Europe, and especially between post-Socialism Eastern European countries and continental western European countries, are quite pronounced. However, given that the cultural and linguistic characteristics within the different countries of the Maghreb and, to a smaller extent, sub-Saharan Africa, are quite homogeneous, this may be less of an issue for the latter regions. Moreover, by analyzing immigrant women from the Maghreb, sub-Saharan Africa and Europe, this study analyzes the largest groups of immigrants in France's immigrant population; it also succeeds in differentiating between immigrants from poor and less developed countries, immigrants from culturally conservative and mostly Muslim countries, and immigrants from more developed countries.

^6^The data is not sufficient to allow for an examination the effect of primary worker status on labor market integration. Instead, I assume that married women, and especially those with children, are the “tied-mover” by default (Raghuram, 2004). Since 1974, immigration to France is permitted only on a family reunification basis (except for immigrants from within the “Schengen” borders). It is thus logical to assume that immigrant women immigrating to France after 1974 from origin countries outside the EU will be tied movers. I am aware that this is not uniformly true. However, as this is often the case and due to the lack of data enabling the identification of primary movers, a large number of studies within the family investment literature adopt these assumptions (Baker and Benjamin, 1997; Raghuram, 2004; Sorenson and Dahl, 2016).

^7^While a strong negative motherhood effect is likely to occur for first-generation immigrant women, this might be not the case for second-generation immigrant women. Second-generation immigrant women are presumed to be proficient in French and be more familiar with French culture compared to the first-generation. They are also more likely to have a family network that can help with childcare and to have developed peer or professional network, which can influence their employment outcomes. Therefore, motherhood is prone to affect second-generation immigrant mothers in a different manner than how it affects first-generation immigrant mothers.

^8^Immigrant mothers from more developed countries can be less inclined to be in employment if they emigrate with a potentially high earning partner and women emigrating from less developed countries may be more inclined to be employed, as the potentially low income of their partner forces them to compensate for his insufficient earnings. Unfortunately, the data provide no information on the partner employment patterns or income, there they effect on labor market outcomes of immigrant women cannot be examined.

^9^I was not able to empirically differentiate the effect of the country of origin's level of development from the effect of the country of origin's level of traditionalism or ideology concerning gender role. However, it is important to address these two approaches, as they are likely to impact the labor market outcomes of immigrants in a different manner. Empirically, these two approaches should be considered competing hypotheses.

^10^Traditional countries' refers to countries with traditional gender role norms, where women's labor participation rates are low, primarily Muslim countries.

^11^In 2010, 2011, 2012 the dataset included 5 values for the variable country of birth: 1. France, 2. Maghreb, 3. Sub-Sahara Africa, 4. Europe, 5. Other countries.

^12^I am not able to control for years since immigration for the immigrant population, since the models of both the native population and the immigrant population need to be analyzed in an identical manner to allow comparison of the interaction between immigration status and motherhood status. Thus, assuming that the variable years since immigration were to be added in the model of the immigrant population, the juxtaposition would become impossible.

^13^I am aware that there might be potential selection bias into motherhood. Selection into motherhood might be different across immigrant groups, and some groups of women might tend to have children at higher rates and at younger ages. Work choices of women are strongly influenced by gender norms, which are likely to differ across countries (Blau and Kahn, 2007; Budig et al., 2012, 2016). Unfortunately, I am not able to take into account the selection into motherhood as the variables present in my data are missing detailed information on motherhood such as children ever born to a woman, age at first birth, and hence, do not allow me to predict selection into motherhood. However, the literature has pointed to the importance of education in shaping fertility patterns (Kahn and Whittington, 1996; Cohen and Blanchi, 1999), therefore controlling for years of education and choosing to perform a regression analysis for immigrant and native women, as well as, for each region of origin separately, might potentially moderate the selection bias into motherhood.

^14^A Robustness check testing the effect of having children regardless of their age is presented in Appendix B.

^15^Unfortunately, I have not been able to take into account the selection into motherhood, as the variables present in my data are missing details on motherhood such as children ever born to a woman, age at first birth, which may vary across immigrant groups. However, the literature has pointed to the importance of education in shaping fertility patterns (Tienda and Glass, 1985; Kahn and Whittington, 1996; Cohen and Blanchi, 1999), therefore controlling for years of education and choosing to perform a regression analysis for immigrant and native women, as well as for each region of origin separately, might potentially moderate the selection bias into motherhood.

^16^Children of the women interviewed living in the household.

^17^The information in the data includes the number of children only by three categories of age—under 3 years old, under 6 years old and under 18 years old. Thus, I cannot analyze the number of children and their age separately. I therefore chose to create a variable that includes having children or not by two categories; below age 6 and between 7 and 18. Moreover, taking into account children under 18 years old allows us to portray longer time effects of motherhood on wages.

^18^The square root term of working hours, was analyzed in order to reduce skewness, however the square root term had more skewness than the natural term (see Appendix A), therefore, I chose to keep the weekly hours as the control variable.

^19^The variables take into account the marital status and not the cohabitation status since I lack cohabitation data such as relationship length. Moreover, since 1974, immigration to France is permitted only on a family reunification basis (except for immigrants from within the “Schengen” borders). Therefore, controlling for marital status instead of cohabitation seems to be much more relevant for immigrant women.

^20^In order to determine if the immigrant population (foreign born) bears a disproportionate negative motherhood effect compared to native mothers, the regression coefficient of motherhood of the foreign-born group will be compared to the coefficient of the native-born group. Whether the differences between the coefficients of motherhood in the regression models for each group are statistically significant will be tested using the following equation:

^21^The predicted probabilities are for 37 year old women since this is the average age of my sample.

^22^Full-time weekly working hours in France.

^23^The illustration of the result will be presented for women working in low white-collar occupations since the largest rate of women work in that kind of occupation in the French labor market.

^24^Native women have 11.36 years of education on average, immigrants from Europe 10.41, and immigrants from the Maghreb and sub-Sahara have 8.45 years of education and 8.93 years of education respectively. Therefore, I chose to illustrate the different profiles of women by the average years of education across groups−10 years of education.

^25^As the aim of this study is to analyze the effect of motherhood on immigrants compared to the effect of motherhood on native women, I decided to include the entire native-born population in the native-born group, regardless of their immigration background (second and third generation of immigrants). Moreover, the second generation are likely to have lower motherhood penalty compared to first-generation immigrant mothers as second-generation immigrant women are supposed to be proficient in French and be more familiar with French culture compared to the first-generation. They are also likely to have developed family or professional networks, which can influence their employment outcomes in a different manner than first-generation immigrant mothers.

^26^Foreign-born women from Maghreb are the group with the highest rate of married women (72%) followed by immigrant women from Europe. Immigrants from sub-Saharan Africa have one of the lowest rates of married women (47%). Immigrants from Maghreb are the least academic group, with only 8.45 years of education on average followed by immigrants from sub-Saharan Africa (8.93). The age ranges from 36.63 years old (among immigrants from sub-Sahara) to 38.56 years old (among the immigrant women from Europe) which may signify more experience for immigrants from Europe, since age can be considered a proxy for experience. Immigrant women from Europe are the group that works the highest number of weekly hours, after which are the native-born women (33.45); the first-generation immigrant women from sub-Sahara are the group working the fewest weekly hours (32.37).

^27^The difference between the effects of working in high blue and low blue-collar types of occupation on the first generation compared to the native population is not statistically significant. The differences in the effect of marriage on the first generation compared to the native population is not statistically significant either. However, the differences in the effect of working in a high white-collar type of occupation on immigrants compared to native-born is statistically significant and adds much more to the yearly income of immigrant women when selection is not controlled for. Consistently with previous studies, years of education affect the immigrant population much less than the native population and each year of education among the native population adds 5% to the yearly wages compared to only 2.2% among the immigrant population. The difference in the effect of weekly working hours is significant as well, and each hour worked weekly is more beneficial to the first-generation immigrant population than to the native population. The effect of age is stronger for the native population which could significate that working experience adds much more to the income of native-born population than to the income of immigrants.

^28^The comparison between natives and immigrants on the effect of marriage, years of education, age and weekly worked hours is significant for the corrected model.

^29^Cukrowska-Torzewska and Lovasz (2020) reported a motherhood penalty of 4.5% per child while Budig et al. (2016) reported a motherhood penalty of 5%. It should be noted, however, that most studies analyzed the motherhood penalty for each child (number of children) or for having children or not and not for having children in two categories of age such as in the current study.

^30^For the sake of parsimony and in order to ease the comparison between the different groups of immigrant and native women, the native Model analyzed in the previous section is added to Table 4.

^31^The results for the two-step selection model were not statistically significant for children between 7 and 18 years old among immigrants from Europe but were statistically significant for children under 6 years old and reveal a greater motherhood premium for young children compared to the uncorrected model. The two-step selection model reveals a similar effect as the uncorrected model for children between 7 and 18 years old among immigrants from the Maghreb. However, the effect of children under 6 years old for immigrants from the Maghreb becomes statistically not significant in the corrected model. The effect of children between 7 and 18 years old on immigrants from sub-Sahara remains statistically not significant when correcting for selection. However, it should be noted that while the effect of children under 6 years old was statistically significant in the uncorrected models, this effect becomes statistically insignificant in the corrected selection model. In the corrected models, the differences between the coefficients of the native-born population and the immigrant population becomes not significant for children under 18 across all groups. The differences between the effect of children for natives and immigrants are significant only for children under 6 years old between natives and first-generation immigrants from Europe. The Lambda term of the Heckman models is negative for all immigrant groups (although it is statistically not significant).

^32^The effect of marriage on the immigrant population compared to the native population is not statistically significant across all groups. The effect of age is statistically significant between natives and all immigrants groups. The comparison of the effect of years of education between native and immigrants is statistically significant across all immigrant origin groups, and years of education affect the immigrant population much less than the native-born population. As a matter of fact, compared to the native-born population, each year of education has the highest positive consequence on the annual income of first-generation immigrants from Maghreb, and the lowest positive consequence on first-generation immigrants from sub-Sahara.

^33^The graphs are based on the uncorrected models since the Lambda term were not significant in any one of the corrected for selection models.
