# Supplementary material for: The Motherhood Penalty of Immigrants in France: Comparing the Motherhood Wage Penalty of Immigrants From Europe, the Maghreb, and Sub-Sahara With Native-Born French Women
Source: Front Sociol. 2022 Mar 30;7:748826. doi: 10.3389/fsoc.2022.748826 (PMC9006993; doi:10.3389/fsoc.2022.748826)
Supplement: Supplementary file 1 [file Data_Sheet_1.docx]

***Appendix A: weekly working hours skewness test***

| Descriptives |  |  |  |  |
| --- | --- | --- | --- | --- |
|  |  |  | Statistic | Std. Error |
| Weekly working hours | Mean |  | 34.66 | 0.041 |
|  | 95% Confidence Interval for Mean | Lower Bound | 34.58 |  |
|  |  | Upper Bound | 34.74 |  |
|  | 5% Trimmed Mean |  | 34.59 |  |
|  | Median |  | 35 |  |
|  | Variance |  | 114.882 |  |
|  | Std. Deviation |  | 10.718 |  |
|  | Minimum |  | 0 |  |
|  | Maximum |  | 100 |  |
|  | Range |  | 99 |  |
|  | Interquartile Range |  | 9 |  |
|  | Skewness |  | 0.443 | 0.009 |
|  | Kurtosis |  | 4.428 | 0.019 |
| SQRT of weekly working hours | Mean |  | 5.8051 | 0.00372 |
|  | 95% Confidence Interval for Mean | Lower Bound | 5.7978 |  |
|  |  | Upper Bound | 5.8124 |  |
|  | 5% Trimmed Mean |  | 5.8484 |  |
|  | Median |  | 5.9161 |  |
|  | Variance |  | 0.96 |  |
|  | Std. Deviation |  | 0.97999 |  |
|  | Minimum |  | 0.55 |  |
|  | Maximum |  | 9.98 |  |
|  | Range |  | 9.43 |  |
|  | Interquartile Range |  | 0.77 |  |
|  | Skewness |  | -0.907 | 0.009 |
|  | Kurtosis |  | 3.693 | 0.019 |

***Appendix B***

**Table 1B**: Robustness check, linear regression predicting the effect of having children according to immigration status and motherhood.


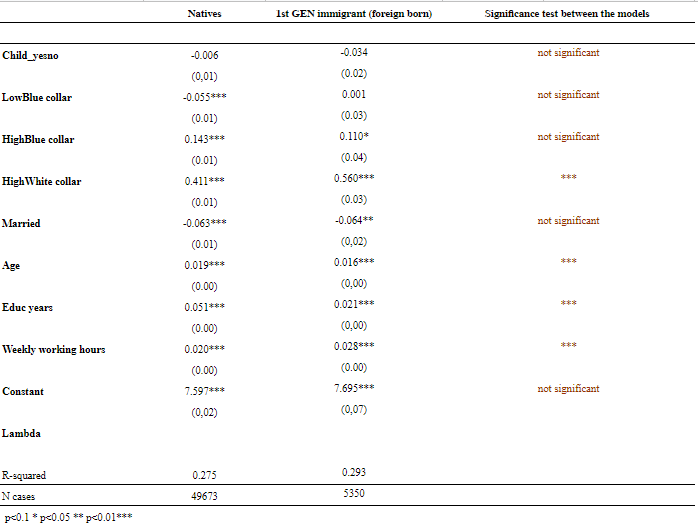


SOURCE: Enquête sur les revenus fiscaux et sociaux (ERFS),INSEE 2009-2012

**Table 2B**: Robustness check, linear regression predicting the effect of having children according to region of origin and motherhood.


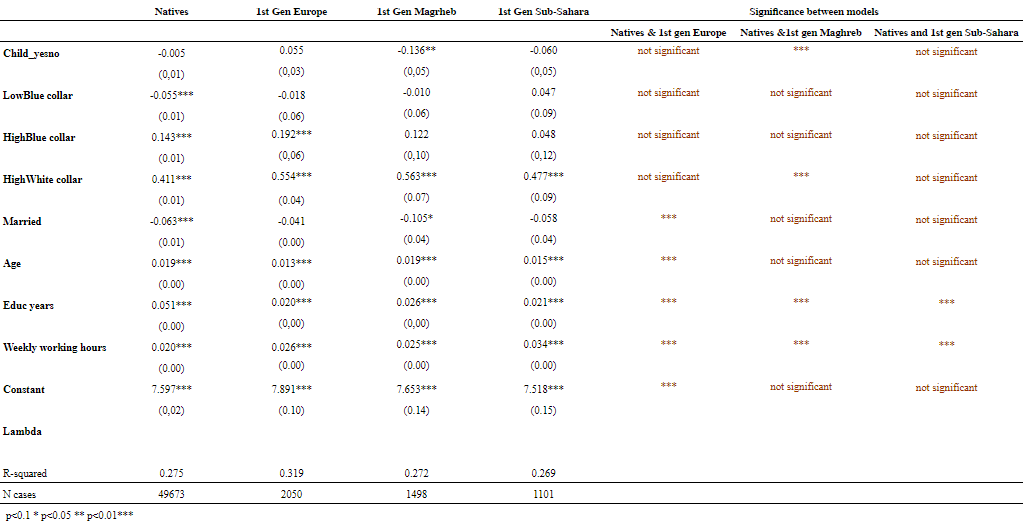


SOURCE: Enquête sur les revenus fiscaux et sociaux (ERFS),INSEE 2009-2012
